# Towards Heart Rate Estimation in Complex Multi-Target Scenarios: A High-Precision FMCW Radar Scheme Integrating HDBS and VLW

**DOI:** 10.3390/s25247629

**Published:** 2025-12-16

**Authors:** Xuefei Dong, Yunxue Liu, Jinwei Wang, Shie Wu, Chengyou Wang, Shiqing Tang

**Affiliations:** 1 School of Physics and Electronic Information, Yantai University, Yantai 264005, China; dongxuefei@s.ytu.edu.cn (X.D.); wushie@ytu.edu.cn (S.W.); tangshiqing@s.ytu.edu.cn (S.T.); 2 School of Airspace Science and Engineering, Shandong University, Weihai 264209, China; wangchengyou@sdu.edu.cn

**Keywords:** frequency-modulated continuous wave (FMCW), vital sign monitoring, multi-target, multiple-input multiple-output (MIMO), fast iterative interpolation beamforming (FIIB)

## Abstract

Non-contact heart rate estimation technology based on frequency-modulated continuous wave (FMCW) radar has garnered extensive attention in single-target scenarios, yet it remains underexplored in multi-target environments. Accurate discrimination of multiple targets and precise estimation of their heart rates constitute key challenges in the multi-target domain. To address these issues, we propose a novel scheme for multi-target heart rate estimation. First, a high-precision distance-bin selection (HDBS) method is proposed for target localization in the range domain. Next, multiple-input multiple-output (MIMO) array processing is combined with the Root-multiple signal classification (Root-MUSIC) algorithm for angular domain estimation, enabling accurate discrimination of multiple targets. Subsequently, we propose an efficient method for interference suppression and vital sign extraction that cascades variational mode decomposition (VMD), local mean decomposition (LMD), and wavelet thresholding (WT) termed as VLW, which enables high-quality heartbeat signal extraction. Finally, to achieve high-precision and super-resolution heart rate estimation with low computational burden, an improved fast iterative interpolated beamforming (FIIB) algorithm is proposed. Specifically, by leveraging the conjugate symmetry of real-valued signals, the improved FIIB algorithm reduces the execution time by approximately 60% compared to the standard version. In addition, the proposed scheme provides sufficient signal-to-noise ratio (SNR) gain through low-complexity accumulation in both distance and angle estimation. Six experimental scenarios are designed, incorporating densely arranged targets and front-back occlusion, and extensive experiments are conducted. Results show this scheme effectively discriminates multiple targets in all tested scenarios with a mean absolute error (MAE) below 2.6 beats per minute (bpm), demonstrating its viability as a robust multi-target heart rate estimation scheme in various engineering fields.

## 1. Introduction

Vital signs are critical indicators for evaluating human health, including body temperature, blood pressure, respiratory rate, and heart rate (HR) [1]. With the rising prevalence of chronic diseases, real-time monitoring of respiratory and heart rates has become increasingly essential for safeguarding lives [2]. Most existing vital sign detection technologies are contact-based, with typical examples including electrocardiography (ECG), photoplethysmography (PPG), and electroencephalography (EEG) [3]. Although these methods offer high accuracy, they are often invasive and inconvenient for continuous or long-term use. As a result, the development of non-contact vital sign detection systems has gained significant importance in healthcare applications [4].

Among various non-contact approaches, radar technology has emerged as a research hotspot due to its electromagnetic sensing advantages in vital sign detection. The primary radar types employed in this domain include continuous wave (CW) radar [5], impulse radio ultra-wideband (IR-UWB) radar [6] and frequency modulation continuous wave (FMCW) radar [7]. CW radar estimates target velocity by analyzing the phase shifts of reflected signals. However, it cannot simultaneously measure distance, which limits its applicability. In contrast, IR-UWB radar supports distance measurement, enabling the discrimination of multiple targets. Nevertheless, this advantage comes at the cost of increased hardware complexity [8]. Compared with above radars, the FMCW radar not only inherits the velocity measurement capability of CW radar but also provides accurate range estimation [9]. Thus, it provides efficient support for multi-target vital sign detection, particularly in the field of HR detection—a persistent technical challenge due to weak signals and susceptibility to interference.

In multi-target FMCW radar vital sign detection, targets are localized and separated based on range and angular information, enabling individual HR estimation for each isolated subject [10]. In [11,12], accurate multi-target localization was achieved by estimating both distance and direction of arrival (DOA), but there was no further separation of the multi-target to estimate the target’s vital sign parameters. Eder et al. [13,14] proposed a dictionary-based approach for multi-target localization and HR estimation, but the high HR estimation accuracy was obtained at a high signal-to-noise ratio (SNR=0dB). In [15,16], range bins corresponding to range-discrete Fourier transform (range-DFT) spectral peaks were directly utilized as final target distance estimates. However, when targets are in close radial proximity, their echo signals exhibit frequency-domain overlap. This spectral aliasing generates a single false peak, resulting in the misidentification of multiple targets as a single object. When multiple targets are located at the same distance from the radar, angle-fast Fourier transform (angle-FFT) is employed to obtain the azimuth angle information of targets [17,18]. However, the angular resolution of angle-FFT is limited and it is susceptible to spectrum aliasing, which affects the accuracy of DOA estimation. Shiraki et al. [12] proposed a method of multiplying the multiple signal classification (MUSIC) spectrum calculated from all combinations of transmitting and receiving stations to improve target localization. C. A. Schroth et al. [19] presented a complete signal processing chain for radar-based multi-target detection and 2D-MUSIC-based DOA estimation. While MUSIC improves resolution compared to DFT-based approaches, it typically involves eigenvalue decomposition and grid search over the spatial spectrum, resulting in a heavy computational burden.

Once target localization is complete, conventional methods typically employ bandpass filters for frequency-domain decomposition to isolate heartbeat components [20]. In practice, however, phase signals are susceptible to various interferences (e.g., respiratory harmonics, intermodulation products, and environmental noise), which leads to a significantly low SNR for heartbeat signals. These interferences become particularly pronounced in multi-target scenarios, severely degrading detection accuracy. To address this, variational mode decomposition (VMD) has been applied in [21,22] to extract heartbeat signals. Nevertheless, standard VMD relies on manual parameter selection, resulting in inconsistent interference suppression in engineering applications [23,24]. Although Huo et al. [25] proposed an SSA-VMD technique to reconstruct vital signs, this approach fails to effectively resolve the aforementioned issues and introduces an extremely high computational burden.

When estimating the frequency of the extracted heartbeat signals to obtain the target’s HR, it is mostly achieved through conventional FFT spectral analysis and peak searching [26,27]. However, heartbeat signals are typically contaminated by respiratory harmonics, intermodulation products, and various noise, whose spectral components often lie close to the fundamental frequency of the heartbeat. In such cases, FFT struggles to achieve accurate frequency estimation due to insufficient frequency resolution and spectral leakage issues.

In recent years, significant advancements have been made in radar-based vital sign monitoring, particularly with the emergence of deep learning (DL) techniques and advanced commercial radar systems. DL-based methods, such as convolutional neural networks (CNNs) and transformers, have demonstrated remarkable accuracy in extracting heart rates from noisy signals [28,29]. However, these data-driven approaches typically require massive annotated datasets for training and impose a heavy computational burden (often requiring GPUs), which challenges their deployment on resource-constrained edge devices. Furthermore, they often lack physical interpretability. Commercial radar solutions have also evolved [30], but they often rely on generic algorithms that lack the flexibility to distinguish closely spaced or occluded targets in complex blind monitoring scenarios.

In the context of this work, specific scenarios of interest are defined to better contextualize the addressed challenges. Closely spaced targets refer to scenarios where multiple subjects are located at approximately the same radial distance from the radar with a narrow angular separation. A typical real-world example is two people sitting side-by-side on a sofa, where high angular resolution is required for effective separation. In contrast, occluded targets refer to front-back arrangement scenarios where the radar’s line-of-sight to the rear target is partially or fully blocked by the front subject. A common instance is students sitting in rows in a classroom, where the chest wall of the rear subject is obscured by the person in front, significantly increasing the difficulty of target signal detection.

To improve target discrimination in occlusion scenarios and enhance HR estimation accuracy, this paper proposes a high-precision measurement framework for multi-target HR estimation. While primarily designed for precise vital sign monitoring, this framework is particularly effective in handling the complexities of distinguishing targets in scenarios with close proximity and front-back occlusion. It achieves this by ensuring interference suppression and super-resolution frequency measurement. The main contributions of this paper are as follows:

1. Comprehensive system architecture: We propose a holistic FMCW radar-based HR estimation framework that integrates distance, angle, and frequency domain processing. This architecture effectively addresses the challenges of distinguishing closely spaced and occluded targets, providing more robust performance compared to traditional methods in complex environments.
2. Target localization strategy: Within this framework, we introduce a high-precision distance bin selection (HDBS) method. Unlike traditional peak-searching methods, this module combines ordered statistics constant false alarm rate (OS-CFAR) detection and density-based spatial clustering of applications with noise (DBSCAN) to effectively identify the distance between the target and the radar.
3. Novel signal enhancement technique: To ensure high precision under low-SNR conditions, the framework proposed a novel interference suppression method termed VLW (cascading VMD, local mean decomposition (LMD), and wavelet thresholding (WT)), this technique effectively isolates high-quality heartbeat components from complex physiological and environmental interferences.
4. Efficient super-resolution frequency estimation: An improved fast iterative interpolated beamforming (FIIB) algorithm is proposed to achieve high-precision heart rate estimation. By leveraging the conjugate symmetry of real-valued signals, this algorithm reduces the computational burden by approximately 60% compared to the standard version while maintaining super-resolution performance.

The rest of this paper is organized as follows: Section 2 reviews the basic principles of FMCW radar vital sign detection, and then multiple-input multiple-output (MIMO) model is described. Our proposed scheme and the performance of the proposed methods are presented in Section 3. Section 4 provides experimental results and their analysis. The conclusion and the further work are given finally in Section 5.

## 2. FMCW Radar Vital Sign Detection Principle

This section introduces the critical intermediate frequency (IF) signal model in FMCW radar-based vital sign detection, as well as the 3-D matrix model of multi-target vital sign signals obtained by MIMO technology. This mathematical formulation lays the necessary theoretical foundation for the multi-target HR estimation scheme proposed in the subsequent section.

### 2.1. Radar Signal Model

**Figure 1:**
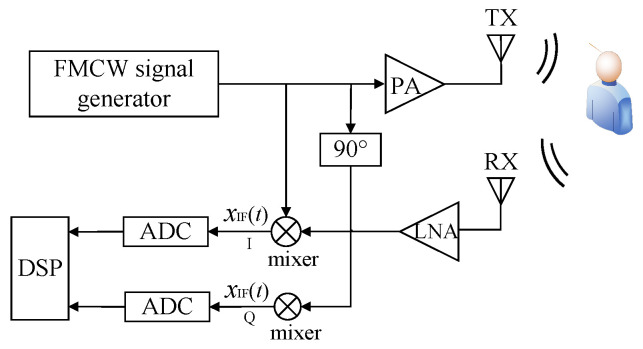
Block diagram of the FMCW radar system.

Figure 1 illustrates the fundamental architecture and operation principle of the FMCW radar system for vital sign detection. The core process involves transmitting a linearly modulated chirp signal, which reflects off the target to form an echo signal. The received echo is then mixed with the local oscillator (LO) signal and a 90° phase-shifted version of the LO signal, respectively, directly yielding the orthogonal I and Q IF signals. Subsequently, these components are digitized by an analog-to-digital converter (ADC) for digital signal processing (DSP).

In the FMCW radar system, the chirp signal $x_{T}\left(t\right)$ from the signal generator is transmitted via the transmitting antenna (TX); it can be depicted as Equation (1):

(1) $$x_{T}\left(t\right)=A_{T}\cos\left(2\pi f_{c}t+\pi \frac{B}{T_{d}}t^{2}+\varphi \left(t\right)\right)$$

where $A_{T}$, $f_{c}$, and $\varphi \left(t\right)$ are the amplitude, initial frequency, and initial phase of the transmitted signal, respectively. *B* is the pulse bandwidth, and $T_{d}$ is the duration of each chirp signal.

After reflecting from the human chest cavity, the received signal $x_{R}\left(t\right)$ at the receiving antenna (RX) can be written as Equation (2):

(2) $$x_{R}\left(t\right)=A_{R}\cos\left(2\pi f_{c}\left(t-t_{d}\right)+\pi \frac{B}{T_{d}}{\left(t-t_{d}\right)}^{2}+\varphi \left(t-t_{d}\right)\right)$$

where $A_{R}$ is the amplitude of the received signal and $t_{d}=2r\left(t\right)/c\;$ is the range-dependent time delay from a given target at radial range $r\left(t\right)$, with *c* being the speed of light.

Thereafter, the IF signal is obtained after the mixing operation of the transmitted signal and the received signal. Then, the complex form expression of the IF signal $x_{\mathrm{IF}}\left(t\right)$ can be expressed as Equation (3):

(3) $$x_{\mathrm{IF}}\left(t\right)=A_{\mathrm{IF}}\exp\left(j\left(2\pi f_{\mathrm{IF}}t+\varphi_{\mathrm{IF}}\left(t\right)\right)\right)$$

where $A_{\mathrm{IF}}$ and $f_{\mathrm{IF}}$ are the amplitude and frequency of the IF signal, respectively. $\varphi_{\mathrm{IF}}\left(t\right)$ is the phase of the IF signal; it is given by Equation (4):

(4) $$\varphi_{\mathrm{IF}}\left(t\right)=\frac{4\pi f_{c}r\left(t\right)}{c}=\frac{4\pi f_{c}\left(d_{0}+s\left(t\right)\right)}{c}$$

where $d_{0}$ is the distance between a target and radar, $s\left(t\right)$ is the displacement of the thorax caused by human physiological activities. In this case, the phase change of the IF signal per unit time $\Delta \varphi_{\mathrm{IF}}$ can be depicted as Equation (5):

(5) $$\Delta \varphi_{\mathrm{IF}}=\frac{4\pi f_{c}\Delta s}{c}$$

Therefore, vital sign information from human chest micro-movement can be obtained by detecting phase changes in the IF signal [9]. Specifically, in the proposed signal processing framework, the IF frequency $f_{\mathrm{IF}}$ corresponds to the target’s radial distance $d_{0}$, enabling target localization in the range domain, whereas the phase variations $\varphi_{\mathrm{IF}}\left(t\right)$ within that range bin are analyzed to retrieve the micro-motion information s(t) (vital sign monitoring).

### 2.2. MIMO Model

In multi-target scenarios, distance domain processing provides the simplest method for differentiating targets. However, when multiple targets are at the same distance from a radar, the angular dimension needs to be introduced for further differentiating targets. During DOA estimation, increasing the angular resolution of a radar requires increasing the number of receiver antennas. In contrast, the MIMO technique exploits virtual antenna expansion to enhance the radar’s angular resolution while reducing the requirement for the number of antennas [15,31] and provides a cost-effective way to improve the angular resolution of a radar [32,33].

**Figure 2:**
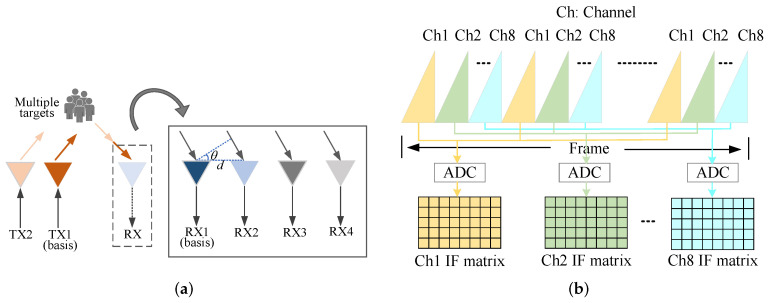
The radar’s configuration and signal matrix. (**a**) Transceiver structure. (**b**) Channel configuration and signal matrix.

In this study, an AWR1642 millimeter-wave radar is employed. Figure 2 illustrates the radar’s transceiver structure, channel configuration, and signal matrix processing flow. Its radio frequency front end is equipped with two TXs (TX1 and TX2) and four RXs (RX1, RX2, RX3, and RX4), as shown in Figure 2a. Through time-division multiplexing (TDM) [27], this configuration can be virtually expanded into an eight-channel receiving antenna array. Within each frame period, the two TXs sequentially emit multiple chirp signals, which are simultaneously received by the four RXs. After undergoing mixing and ADC sampling, each channel obtains an IF signal matrix, as illustrated in Figure 2b.

For the convenience of description, supposing the number of TX and RX of the MIMO radar are $N_{\mathrm{TX}}$ and $N_{\mathrm{RX}}$, respectively. Then the number of channels after the virtual expansion is *I*, and $I=N_{\mathrm{TX}}\times N_{\mathrm{RX}}$. From Equation (3), the IF signal at the *n*th chirp in the *i*th channel of the MIMO radar ${x_{\mathrm{IF}}}^{n,i}\left(t\right)$ can be expressed as Equation (6):

(6) $${x_{\mathrm{IF}}}^{n,i}\left(t\right)=A_{\mathrm{IF}}\exp\left(j\left(2\pi f_{\mathrm{IF}}t+{\varphi_{\mathrm{IF}}}^{n}\left(t\right)+\frac{2\pi d_{i}\sin\theta }{\lambda }\right)\right)$$

where the term $\frac{2\pi d_{i}\sin\theta }{\lambda }$ represents the spatial phase shift induced by the geometric path difference between the *i*-th virtual antenna and the reference antenna. Here, $d_{i}$ denotes the spatial distance between the *i*-th virtual antenna and the reference antenna (typically the first element), θ represents the target’s azimuth angle relative to the radar boresight, and λ is the signal wavelength. After sampling of IF signals, the matrix form of IF signals from multiple targets $X\left(m,n,i\right)$ can be written as Equation (7):

(7) $$X\left(m,n,i\right)=\sum_{l=1}^{L_{T}}A_{\mathrm{IF}}\exp\left(j\left(2\pi f_{{\mathrm{IF}}_{l}}mT_{s}+{\varphi_{n}}_{l}+\frac{2\pi d_{i}\sin\theta_{l}}{\lambda }\right)\right)$$

where m=1,2,…,M, n=1,2,…,N, and i=1,2,···,I. *M* and *N* represent the fast and slow time dimensions, respectively, and *I* is the number of channels. Additionally, $L_{T}$ is the number of targets, and $T_{s}$ is the actual ADC sampling interval. Then, the raw data forms a 3-D matrix of M×N×I. It is important to note that in practical scenarios, the received signal is not ideal; it is inevitably contaminated by static clutter, coupled biological interferences (e.g., respiration harmonics), and system noise. Therefore, effective signal separation and enhancement strategies are required.

## 3. Multiple-Target Heart Rate Estimation Scheme

**Figure 3:**
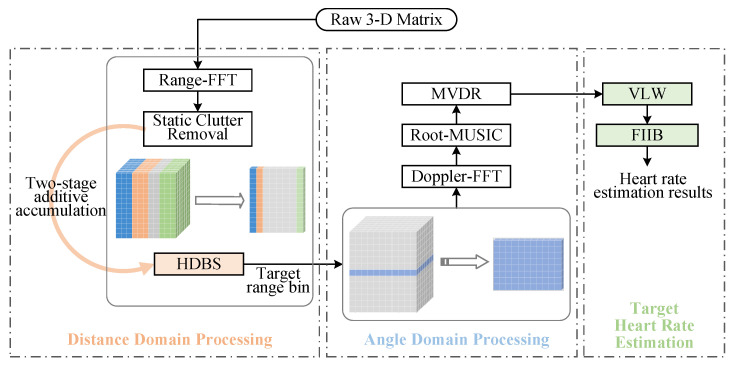
Block diagram of the proposed scheme.

In this study, we propose a novel scheme for multi-target HR estimation based on FMCW radar, as illustrated in Figure 3. First, distance domain processing is performed: the raw 3-D matrix undergoes Range-FFT to generate a range-time map, and static clutter removal is performed to eliminate interference from static objects. On this basis, the SNR of the target is improved through two-stage additive accumulation, and the proposed HDBS algorithm is used for accurate target distance estimation. Next, for targets at the same distance from the radar, angle domain processing is introduced: the Root-MUSIC and minimum variance distortionless response (MVDR) algorithms are utilized to estimate target angles and separate multiple targets. During target HR estimation, the proposed VLW algorithm is employed to effectively suppress interference and extract pure heartbeat signals. Furthermore, the FIIB algorithm is adopted to achieve high-precision and super-resolution vital sign detection with low computational burden.

### 3.1. Distance Domain Processing

#### 3.1.1. Static Clutter Removal

In indoor environments, static objects such as walls and furniture generate strong echo signals (i.e., static clutter) that significantly interfere with the detection of weak vital sign signals. To mitigate this, static clutter removal is performed on the Range-FFT output matrix (range-time map) before further processing. This is achieved by subtracting the mean value of the slow-time samples from each range bin, effectively removing the zero-Doppler components. The process can be mathematically expressed as Equation (8):

(8) $${\hat{R}}_{i}\left[m,n\right]=R_{i}\left[m,n\right]-\frac{1}{N}\sum_{k=1}^{N}R_{i}\left[m,k\right]$$

where ${\hat{R}}_{i}\left[m,n\right]$ denotes the spectral data matrix of the *i*-th channel after static clutter removal, $R_{i}\left[m,n\right]$ is the output of Range-FFT. m=1,2,…,M, where *M* is the number of Range-FFT points; n=1,2,…,N, where *N* represents the number of transmitted radar chirps; and i=1,2,…,I, where *I* is the number of antenna array channels.

#### 3.1.2. Accumulation for Distance Estimation

**Figure 4:**
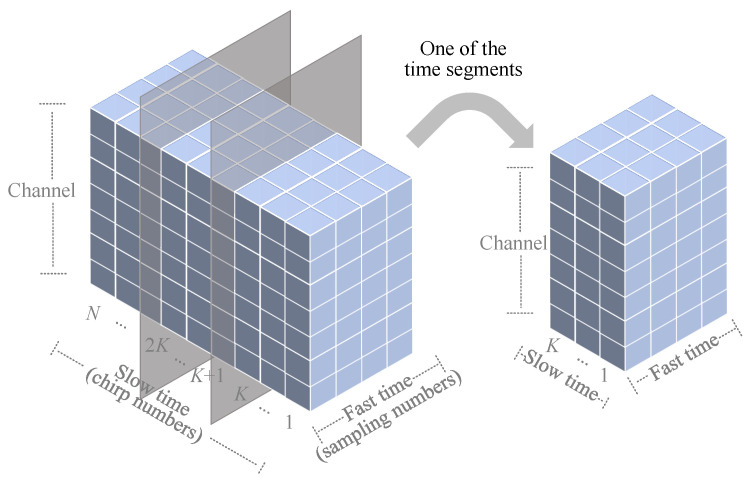
Time segmentation chart.

**Figure 5:**
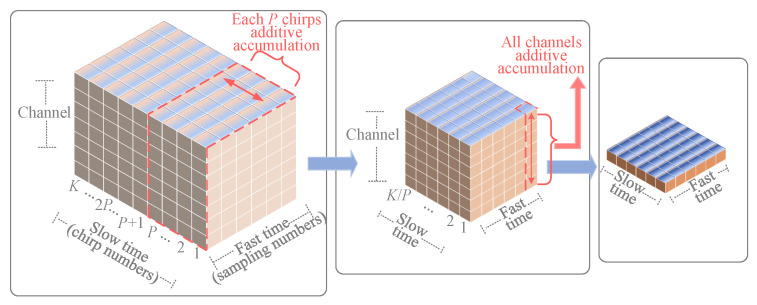
Two-stage accumulation in a time segment.

Considering the temporal accumulation characteristics of the slow-time dimension, as shown in Figure 4, we divide the 3-D data into time segments. Each *K* column is grouped into one time segment, thereby dividing the *N* columns of data into N/K time segments. Then, within each time segment, the 3-D data undergoes two-stage accumulation to achieve significant SNR improvement with low computational complexity, thereby enhancing target detection capability, as shown in Figure 5. The first accumulation sums data across all *I* channels at the same position, converting the original M×K×I structure into an M×K matrix, where *M* and *K* represent the fast and slow time dimensions, respectively. The second accumulation is applied along the slow time dimension by summing every *P* consecutive columns, resulting in K/P columns. After two accumulation operations, the 3-D signal matrix is converted into a 2-D matrix of size M×K/P.

Based on experimental experience, the parameters are set to K=128 and P=8. This configuration aims to provide a theoretical SNR gain of approximately 9 dB, thereby significantly enhancing the visibility of weak targets in low-SNR environments.

#### 3.1.3. Distance Estimation of Targets via HDBS

The conventional method determines the distance between a target and a radar by searching for the range bin corresponding to the peak in the range-FFT spectrum. However, it exposes significant limitations in complex multi-target scenarios such as closely spaced targets or occluded targets, leading to missed detections. In contrast, the OS-CFAR algorithm [34] utilizes adaptive thresholding to identify all range bins exceeding the threshold, thus effectively capturing all target range bins. For these range bins exceeding the threshold, the two-dimensional DBSCAN [35] algorithm can exclude non-target range bins and cluster those belonging to the same target. Compared to K-means clustering, which requires the number of targets to be known a priori, DBSCAN is employed here because it can automatically determine the number of target clusters based on density and effectively identify noise points as outliers, making it more suitable for blind multi-target monitoring. Based on the above two algorithms, we propose a novel range bin estimation algorithm called HDBS, which effectively addresses the traditional method’s limitations in distinguishing close-proximity targets and recognizing occluded targets. It operates on each time segment to identify target range bins through the following steps.

1. Screen potential range bins: The OS-CFAR algorithm employs column-by-column scanning on the M×K/P 2-D matrix to identify all range bins exceeding the threshold.
2. Determine target clusters: The DBSCAN algorithm is applied to determine the target clusters among all range bins that pass the threshold.
3. Define target range bins: Based on the energy characteristics of the target signal in the time dimension, we perform energy aggregation for each cluster. This is achieved by calculating the sum of the squared magnitudes of the complex Range-FFT coefficients along the slow-time dimension for all range bins within the cluster. The range bin with the maximum summed energy is then selected as the final target range bin for the corresponding cluster.

**Figure 6:**
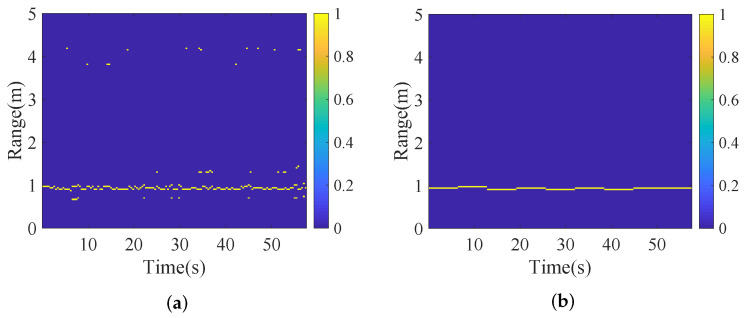
Range-time map. (**a**) Via OS-CFAR. (**b**) Via HDBS.

Figure 6 illustrates the range-time map after the OS-CFAR algorithm and HDBS algorithm for two targets at 1 m. As shown in Figure 6a, following OS-CFAR processing, although the target range bins are successfully detected without omission, a significant number of non-target range bins exceed the detection threshold. In contrast, Figure 6b indicates that by further applying DBSCAN to cluster range bins and selecting target range bins based on maximum energy summation (i.e., the proposed HDBS algorithm), non-target range bins are effectively excluded, enabling precise distance localization. The above results validate the efficacy of the HDBS algorithm for target range bin estimation. The ranging performance of this algorithm is presented in the experiment of Section 4.3.

### 3.2. Angle Domain Processing

#### 3.2.1. Accumulation for Angle Estimation

**Figure 7:**
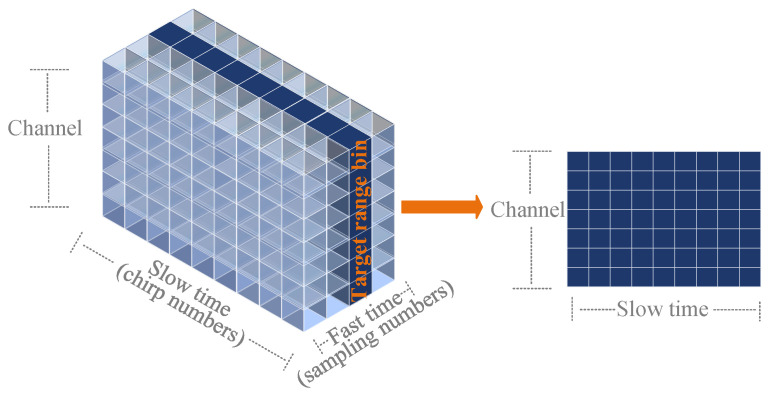
The 2-D matrix corresponding to the target range bin.

To achieve signal accumulation gain at low computational cost while significantly enhancing the SNR, this scheme performs FFT operations along both fast-time and slow-time dimensions, establishing a critical foundation for high-precision angle estimation. As illustrated in Figure 7, the slow-time and channel dimensional data corresponding to the target range bin are extracted, which forms a 2-D matrix of N×I. This matrix is subjected to Doppler-FFT along slow-time, and the operation is repeated *I* times for a time segment (*I* denotes the number of channels), achieving signal accumulation gain through successive Doppler-FFT.

#### 3.2.2. Multi-Target Separation

While the original MUSIC algorithm [36,37] enables high-precision and super-resolution DOA estimation, it suffers from excessive computational complexity. Thus, we use the Root-MUSIC algorithm [38]—which reduces complexity significantly while maintaining comparable accuracy—for DOA estimation on the N×I 2-D matrix (after achieving the accumulation gain) for each time segment. Subsequent target separation employs the MVDR algorithm [39], which suppresses interference by minimizing total array output power while preserving undistorted target signals from the desired direction.

### 3.3. Target Heart Rate Estimation

#### 3.3.1. Heartbeat Signal Extraction via VLW

**Figure 8:**
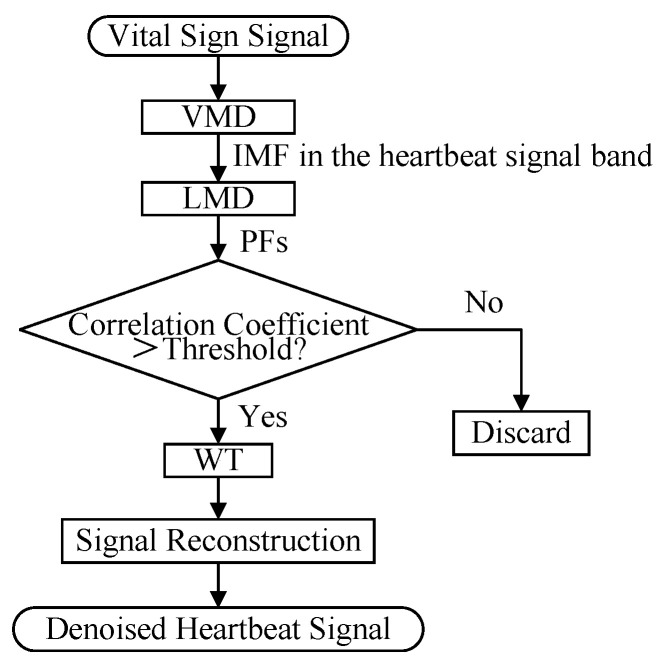
Flowchart of the VLW algorithm.

VMD [40,41] is commonly used to reconstruct heartbeat signals after signal separation, but its interference suppression performance relies heavily on subjective parameter selection in engineering applications. To address this issue, we propose a novel VLW method—cascading VMD, LMD [42], and WT—for enhanced interference suppression after VMD. The cascading order of these three algorithms follows a coarse-to-fine signal processing strategy. First, VMD is employed as a global filter to separate the signal into intrinsic modes, effectively isolating the heartbeat band from strong respiratory interference [43]. Subsequently, LMD performs fine-grained decomposition on the selected mode to extract local features. Finally, WT is applied to remove residual high-frequency noise [42]. This sequential approach ensures that each stage processes a progressively cleaner signal, maximizing feature extraction efficiency. The procedure of the VLW algorithm is illustrated in Figure 8 as follows:

1. VMD is employed to decompose the target vital sign signal into a series of IMFs with distinct characteristics. Subsequently, the IMF component corresponding to the heartbeat signal band is selected.
2. The selected IMF is further decomposed into multiple PF components using LMD. Then, the correlation coefficients between each PF component and the vital sign signal are calculated. Further, PF components with correlation coefficients exceeding a predefined threshold are preserved, while those below the threshold are discarded. Among them, the selection of the predefined threshold is based on experimental experience. We conducted a sensitivity analysis on the dataset by varying the threshold from 0.1 to 0.9. It was observed that a threshold of 0.35 yielded the optimal performance in terms of SNR improvement. A lower threshold tends to include residual noise and respiratory harmonics, whereas a higher threshold may discard certain weak yet valid heartbeat components. Therefore, the threshold was fixed at 0.35 for all experiments in this study. The correlation coefficients $r_{i}$ of a PF component with the original vital sign signal follow the form of Equation (9) [44]:
  (9) $$r_{i}=\frac{\sum_{j=1}^{n}\left[x\left(j\right)-\bar{x}\right]\left[y_{i}\left(j\right)-\bar{y_{i}}\right]}{\sqrt{{\sum_{j=1}^{n}\left[x\left(j\right)-\bar{x}\right]}^{2}{\left[y_{i}\left(j\right)-\bar{y_{i}}\right]}^{2}}}$$
  where $r_{i}$ is the correlation coefficient between the *i*th PF component and the vital sign signal, $i=1,2,\ldots ,I_{k}$, $I_{k}$ is the number of PFs, $x\left(j\right)$ and $y_{i}\left(j\right)$ are the *j*th sampling point of the vital sign signal and the *i*th PF component, respectively. Additionally, $\bar{x}$ and ${\bar{y}}_{i}$ denote the mean value of *x* and $y_{i}$, respectively.
3. For the retained PF components, WT is applied to remove noise [45]. Ultimately, the denoised heartbeat signal is reconstructed using the processed PF components.

**Figure 9:**
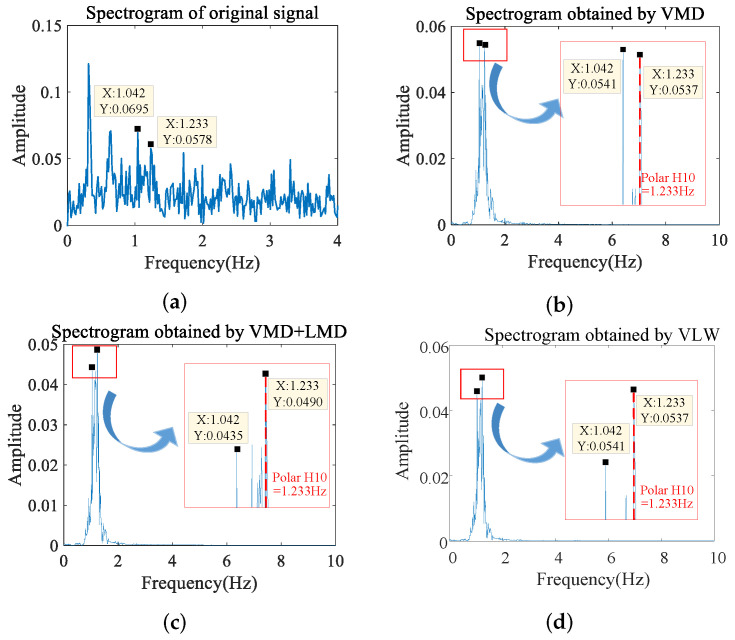
Signal spectrum. (**a**) Original signal. (**b**) VMD processed. (**c**) VMD + LMD processed. (**d**) VLW processed.

To visually demonstrate the progressive signal enhancement, Figure 9 presents the spectrograms at each stage of the proposed VLW framework. As shown in Figure 9a, the original signal spectrum is dominated by strong low-frequency respiratory components (around 0.3 Hz) and significant noise, which completely mask the true heartbeat frequency (1.233 Hz). Figure 9b shows the result after VMD. While the dominant respiratory peak is effectively removed, a strong interference peak at 1.042 Hz (likely a respiratory harmonic) remains prominent, competing with the true heartbeat peak. Figure 9c illustrates the effect of the intermediate VMD + LMD stage. Here, the fine-grained decomposition successfully suppresses the interference peak at 1.042 Hz, making the true heartbeat frequency at 1.233 Hz the dominant component. However, a noticeable noise floor persists. Finally, Figure 9d displays the result of the complete VLW algorithm. After applying wavelet thresholding, the residual random noise is eliminated, resulting in a clean spectrum with a distinct and accurate heartbeat peak. This step-by-step analysis confirms the necessity and effectiveness of the cascaded VMD-LMD-WT structure.

While the spectrograms provide visual confirmation of interference suppression, a quantitative assessment is necessary to rigorously evaluate the estimation accuracy. To achieve this, the mean absolute error (MAE) [46], denoted as $e_{\mathrm{MAE}}$, is utilized as a primary metric. It is formulated as Equation (10):

(10) $$e_{\mathrm{MAE}}=\frac{1}{N_{k}}\sum_{k=1}^{N_{k}}\left|h_{\mathrm{est}}\left(k\right)-h_{\mathrm{true}}\left(k\right)\right|$$

where $N_{k}$ is the total number of time windows in the observation time, and $h_{\mathrm{est}}\left(k\right)$ and $h_{\mathrm{true}}\left(k\right)$ are the estimated and reference values in the *k*th time window, respectively.

**Figure 10:**
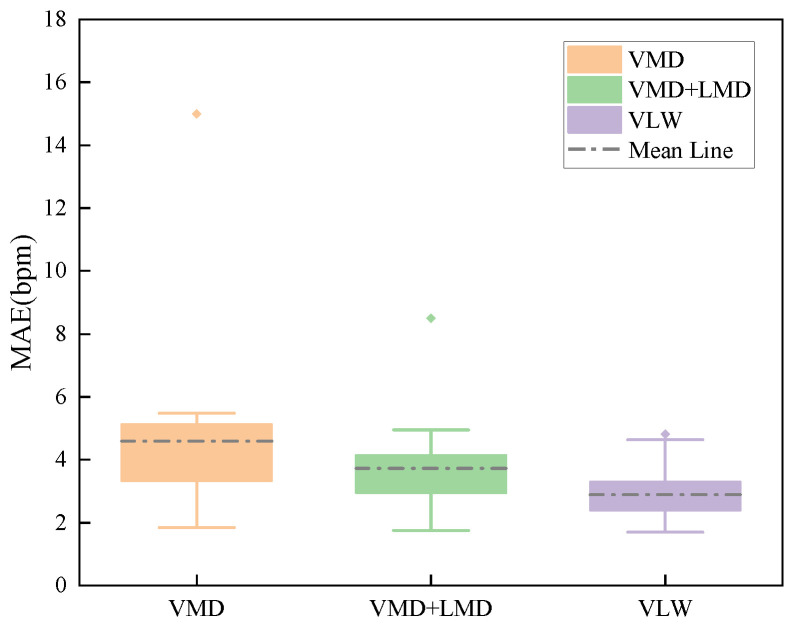
Boxplot formed by MAE of HR using VMD, VMD + LMD, and VLW algorithms, respectively.

Based on this metric, we calculated the HR estimation errors for the heartbeat signals extracted by VMD, the intermediate VMD + LMD stage, and the final VLW algorithm. The statistical results for 20 datasets are presented as boxplots in Figure 10. As observed, the VMD results exhibit significant outliers and a high median error (average MAE = 4.59 bpm), indicating that residual interference often leads to incorrect frequency estimation. The intermediate VMD + LMD stage shows a clear improvement, effectively mitigating severe outliers and reducing the error spread. Finally, the proposed VLW algorithm achieves the best performance with the narrowest distribution, reducing the average MAE to 2.89 bpm. This represents a total accuracy improvement of 1.70 bpm compared to using VMD alone, demonstrating that the complete cascaded structure is essential for robust high-precision estimation.

Finally, the computational cost evaluation of the VLW module is performed on the same laptop with a quad-core Intel Core i5-10210U processor (clock 1.60 GHz) and 8 GB of RAM (Intel Corporation, Santa Clara, CA, USA). The average execution time for processing a data window of 1200 samples (corresponding to approximately 60 s of data) is 0.27 s. This result indicates that the processing speed is significantly faster than the data acquisition rate, demonstrating that the proposed algorithm is computationally efficient and well-suited for real-time monitoring.

#### 3.3.2. Heart Rate Estimation via FIIB

In fact, heartbeat signals are often contaminated by respiratory harmonics, intermodulation, and various types of interference. Since these frequencies may be very close to the fundamental heartbeat frequency, high-precision and high-resolution frequency estimation is crucial. Although algorithms such as MUSIC can achieve precise and high-resolution frequency estimation, their high computational complexity hinders real-time processing. To address this issue, we adopt the FIIB algorithm [47,48,49], which utilizes an iterative and interpolative subtraction strategy to efficiently suppress spectral leakage caused by respiratory harmonics and the fundamental heartbeat components of different targets. Furthermore, the computational complexity of FIIB is optimized, enabling accurate HR estimation with significantly reduced computational cost.

The denoised heartbeat signal $x\left(n\right)$ is modeled as the sum of *L* signals with distinct frequencies, which can be expressed as Equation (11):

(11) $$x\left(n\right)=\sum_{l=1}^{L}A_{l}e^{j2\pi f_{l}n},n=0,1,\ldots ,N_{S}-1$$

where $A_{l}$ is the complex amplitude, $f_{l}$ is the normalized frequency ($f_{l}\in \left[0,\left.1\right)\right.$), and $N_{S}$ is the number of ADC samples per chirp. The flow of the FIIB algorithm is presented in Algorithm 1. In step (i), the heartbeat signal is transformed into the frequency domain by an N-point FFT. Subsequently, the frequency estimation of the heartbeat signal is carried out via coarse estimation and fine estimation. Steps (ii)–(iii) constitute the coarse estimation loop, which runs exclusively during the first iteration (i.e., when q=1), generating *L* coarse estimation of frequency components. Note that ${\hat{S}}_{i}\left(n\right)$ in step (ii) is the DFT coefficient corresponding to the th estimated frequency component ${\hat{f}}_{i}$. That is ${\hat{S}}_{i}\left(n\right)={\left.{\hat{S}}_{i}\left(f\right)\right|}_{f=\frac{n}{N_{S}}}$, where

(12) $${\hat{S}}_{i}\left(f\right)=\sum_{k=0}^{N_{S}-1}e^{j2\pi k{\hat{f}}_{i}}e^{-j2\pi f}=\frac{1-e^{j2\pi N_{S}\left({\hat{f}}_{i}-f\right)}}{1-e^{j2\pi \left({\hat{f}}_{i}-f\right)}}$$

Steps (iv)–(vi) represent the fine estimation phase, where *L* coarse estimation frequencies are refined. Notably, in step (iv), we calculate new leakage-corrected DFT coefficients that are *p* bins away from ${\hat{f}}_{l}$, and the leakage of the other l−1 estimated frequency components is subtracted at this point. That is

(13) $$\begin{array}{cc} {\tilde{X}}_{p}\left({\hat{f}}_{l}\right) & ={\tilde{X}}_{p}\left({\hat{f}}_{l}+\frac{p}{N_{S}}\right)\\ \; & =X\left({\hat{f}}_{l}+\frac{p}{N_{S}}\right)-\sum_{i=1,\;i\neq l}^{L}A_{i}{\hat{S}}_{i}\left({\hat{f}}_{l}+\frac{p}{N_{S}}\right) \end{array}$$

Step (vii) is employed to update the amplitude estimates. The entire algorithm process is run for *Q* iterations in total.    
**Algorithm 1:** FIIB Algorithm Flow
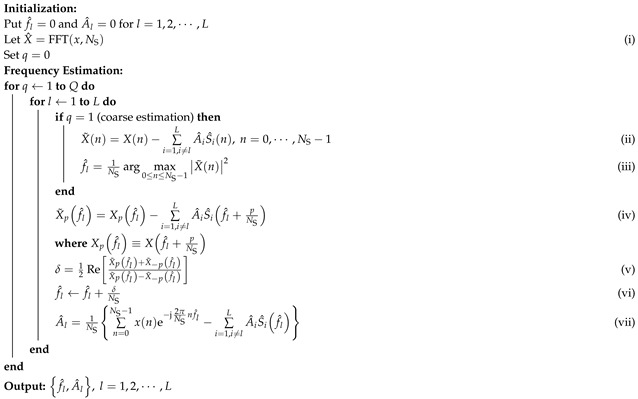


To verify the high-precision frequency measurement capability and super-resolution performance of the FIIB algorithm, this study designed two sets of simulation experiments, with the input signal $x\left(n\right)$ given by Equation (14):

(14) $$x\left(n\right)=A_{1}\sin\left(2\pi f_{1}\frac{n}{f_{s}}\right)+A_{2}\sin\left(2\pi f_{2}\frac{n}{f_{s}}\right),n=0,\ldots ,N_{S}-1$$

where $N_{S}$ is the signal length, $f_{s}$ is the sampling frequency, and $f_{1}$ and $f_{2}$ are the frequencies of components 1 and 2, respectively. Also, $A_{1}$ and $A_{2}$ are the amplitudes of frequency components 1 and 2, respectively. The root mean square error (RMSE), denoted as $e_{\mathrm{RMSE}}$, and the mean square error (MSE), denoted as $e_{\mathrm{MSE}}$, are employed as measurement metrics, and can be depicted as Equations (15) and (16), respectively:

(15) $$e_{\mathrm{RMSE}}=\sqrt{\frac{1}{N_{K}}{\sum_{k=1}^{N_{K}}\left({\hat{f}}_{k}-f_{k}\right)}^{2}}$$

(16) $$e_{\mathrm{MSE}}=\frac{1}{N_{K}}{\sum_{k=1}^{N_{K}}\left({\hat{f}}_{k}-f_{k}\right)}^{2}$$

where $N_{K}$ is the number of Monte Carlo experiments, and ${\hat{f}}_{k}$ and $f_{k}$ denote the estimated and true values of the frequency for the *k*th experiment, respectively. All algorithms are subjected to 5000 Monte Carlo experiments.

(a) Frequency estimation accuracy analysis

To validate the performance of the FIIB algorithm for HR estimation, we configured experimental parameters consistent with those typically used in HR estimation. The experimental parameters were set as $A_{1}=1$, $A_{2}=0.8$, $N_{S}=200$, and a sampling frequency of 20 Hz ($f_{s}=20\;\mathrm{Hz}$), yielding a frequency resolution of 0.1 Hz ($\Delta f=f_{s}/N_{S}=0.1\;\mathrm{Hz}$). To demonstrate the algorithm’s high precision, $f_{1}=1.05\;\mathrm{Hz}$ and $f_{2}=f_{1}+\Delta f=1.25\;\mathrm{Hz}$ were chosen, creating a spacing that is twice the frequency resolution of the FFT.

**Figure 11:**
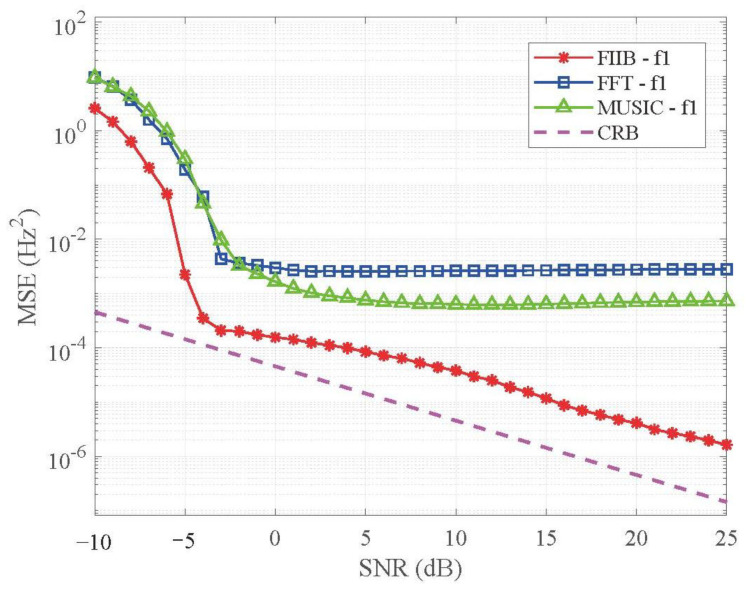
MSE versus SNR for $f_{1}$.

Figure 11 illustrates the MSE versus SNR on a logarithmic scale for the frequency component 1 ($f_{1}$) (the curves for the frequency component 2 are very similar to those of the frequency component 1). It can be observed that the performance of the FFT and MUSIC algorithms saturates at high SNR levels (SNR>−5dB), exhibiting a distinct error floor. This limitation is attributed to the inherent picket-fence effect and the finite search step size imposed by the discrete frequency grid. In contrast, the proposed FIIB algorithm (red curve) successfully breaks through this grid limitation. Its MSE curve continues to decline linearly as the SNR increases, maintaining a slope that is strictly parallel to the Cramer–Rao Bound (CRB). Specifically, at SNR=25dB, the MSEs of FIIB, FFT, and MUSIC are $1.7\times 10^{-6}\;Hz^{2}$, $2.8\times 10^{-3}\;Hz^{2}$, and $7.3\times 10^{-4}\;Hz^{2}$, respectively. This significant quantitative improvement confirms that the FIIB algorithm effectively eliminates grid quantization errors.

The computational cost comparison is performed on a laptop with a quad-core Intel Core i5-10210U processor (clock 1.60 GHz) and 8 GB of RAM (Intel Corporation, Santa Clara, CA, USA). The MUSIC algorithm relies on the eigenvalue decomposition of the covariance matrix and full frequency band spectral peak search, leading to enormous computational complexity. In contrast, FIIB avoids spectral peak search through iterative optimization, significantly reducing the computational burden while maintaining estimation accuracy comparable to that of MUSIC.

(b) Super-resolution capability analysis

In this experiment, to verify the super-resolution performance of FIIB, we set $f_{2}=f_{1}+0.6\Delta f$, and other parameters remain consistent.

**Figure 12:**
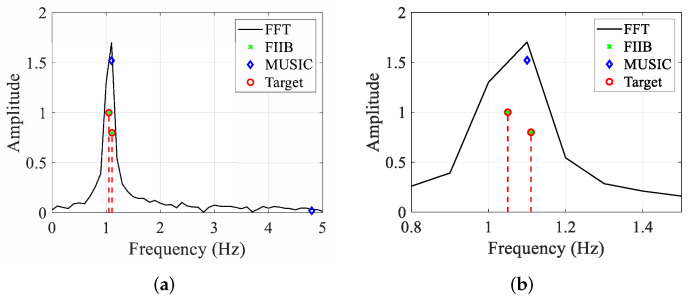
Spectrograms of FFT, FIIB, and MUSIC. (**a**) Full frequency band. (**b**) Partial enlargement in the frequency range [0.8, 1.5].

Figure 12 illustrates the spectrograms generated by FFT, FIIB, and MUSIC when SNR=20 dB. Clearly, the FFT algorithm failed to distinguish these two components, merging them into a single peak due to its limited frequency resolution. Also, MUSIC can only estimate one frequency component and fails to distinguish these two signals, analogous to the resolution limitation in single-snapshot spatial domain processing. In contrast, the FIIB algorithm can precisely estimate these two frequency components, achieving the RMSE of 0.06 Hz for both $f_{1}$ and $f_{2}$. The reason is that FIIB can eliminate spectral leakage by sequentially removing strong frequency components through iterative interpolation when dealing with multiple frequency components, thus achieving high-accuracy and super-resolution frequency estimation.

Overall, FIIB exhibits high-precision estimation and super-resolution capabilities, coupled with low computational complexity, rendering it highly suitable for HR estimation. Additionally, we validated the scalability of the FIIB algorithm by extending the parameter L in the simulation experiments. We tested the algorithm’s robustness with L=6 and L=8 (originally L=4, corresponding to two effective frequency components) under different numbers of frequency components. The experimental results showed that the resulting MSE curves for L=6 and L=8 were statistically indistinguishable from the L=4 baseline curve. This result validates the improved FIIB algorithm’s good scalability and consistency, even with an increase in the number of targets.

The original FIIB algorithm was developed for complex-valued array signals and achieves super-resolution DOA estimation through an iterative interpolation–subtraction strategy. To adapt this for our application, the proposed improved FIIB algorithm capitalizes on the conjugate symmetry of real-valued signals to minimize computational redundancy. Since the extracted phase signal represents physical thoracic displacement, it is inherently a real-valued time series. Consequently, its frequency spectrum exhibits strict conjugate symmetry, where the DFT coefficients satisfy $X\left(n\right)=X^{*}\left(N_{S}-n\right)$, making this optimization universally applicable to all standard radar-based vital sign monitoring scenarios. Directly applying the original FIIB algorithm to the full frequency spectrum would therefore lead to redundant computations.

To address this issue, the proposed FIIB algorithm constrains the core iterative estimation process—including frequency-domain search, interpolation, and amplitude correction—to the positive frequency band. Since the input signal is real-valued, the number of frequency components *L* is even, ensuring a one-to-one conjugate correspondence between positive and negative frequencies. Only L/2 frequency components and their corresponding complex amplitudes are estimated, while the negative-frequency components are reconstructed based on the conjugate relationship ${\hat{A}}_{L-l+1}={{\hat{A}}_{l}}^{*}$ and ${\hat{f}}_{L-l+1}=1-{\hat{f}}_{l}$, where l=1,2,…,L/2, effectively reducing computational complexity.

Specifically, the interference cancellation in step (ii) of the original FIIB algorithm is formulated as Equation (17):

(17) $$\tilde{X}\left(n\right)=X\left(n\right)-\sum_{i=1,i\neq l}^{L}{\hat{A}}_{i}{\hat{S}}_{i}\left(n\right),\;n=0,\ldots ,N_{S}-1$$

Since the components of a real-valued signal form conjugate-symmetric pairs, Equation (17) can be explicitly expanded over positive and negative frequencies as shown in Equation (18):

(18) $$\tilde{X}\left(n\right)=X\left(n\right)-\sum_{i=1,i\neq l}^{L/2\;}{\hat{A}}_{i}{\hat{S}}_{i}\left(n\right)-\sum_{k=L/2\;+1,k\neq l}^{L}{\hat{A}}_{k}{\hat{S}}_{k}\left(n\right),\;n=0,\ldots ,N_{S}-1$$

where the indices $i\in \left[1,L/2\;\right]$ and $k\in \left[L/2\;+1,L\right]$ correspond to the positive and negative frequency components, respectively. For real-valued signals, the *k*th component is the conjugate symmetric counterpart of the *i*th component, i.e., for a pair of components where $f_{k}=1-f_{i}$, their complex amplitudes satisfy ${\hat{A}}_{k}{\hat{S}}_{k}\left(n\right)={{\hat{A}}_{i}}^{*}{{\hat{S}}_{i}}^{*}\left(n\right)$. Hence, the interference from a conjugate pair can be combined as Equation (19):

(19) A^iS^in+A^kS^kn=2ReA^iS^in

Consequently, Equation (18) can be rewritten for the improved FIIB algorithm as Equation (20):

(20) X˜n=Xn−∑i=1,i≠lL/22ReA^iS^in,n=0,…,NS/2−1

Similarly, steps (iv) and (vii) of the improved FIIB algorithm can be further derived as Equations (21) and (22), respectively:

(21) X˜pf^l=Xpf^l−∑i=1,i≠lL/22ReA^iS^if^l+pNS

(22) A^l=1NS∑n=0NS/2−1xne−j2πNSnf^l−∑i=1,i≠lL/22ReA^iS^if^l

The complete procedure is summarized in Algorithm 2. Specifically, given that the vital sign signal is real-valued, its spectrum exhibits Hermitian symmetry (i.e., the negative frequency component is the complex conjugate of the positive one). Leveraging this property, the proposed algorithm performs target searching and iterative estimation only within the positive frequency band. Crucially, the spectral leakage contributions from both positive and negative frequencies are analytically reconstructed and simultaneously removed during each frequency refinement step (steps (ii), (iv), and (vi)). This design reduces the computational complexity by at least 50%, while fully preserving the leakage correction capability and maintaining the high accuracy and super-resolution performance of the original FIIB. As a result, the improved scheme achieves significantly enhanced overall efficiency.    
**Algorithm 2:** Improved FIIB Algorithm Flow
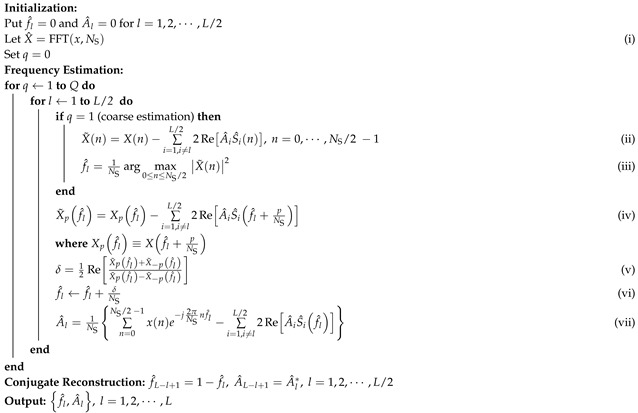


To validate the performance of the improved FIIB algorithm in terms of computational efficiency and convergence behavior, a series of simulation experiments were conducted and compared with the original FIIB algorithm. All experimental parameters were consistent with those in simulation experiment (b) of Section 3.3.2.

**Figure 13:**
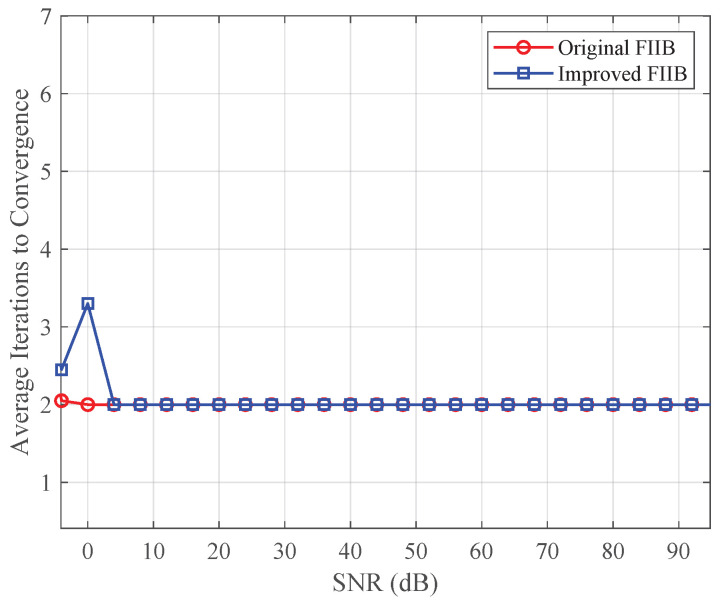
Average number of iterations to convergence for the original and improved FIIB algorithms versus SNR.

Figure 13 illustrates the number of iterations required for the original and improved FIIB algorithms to reach convergence under different SNR conditions, where convergence is defined as an estimation error between two consecutive iterations smaller than 0.001. When the SNR is below 4 dB, we observe that the improved FIIB algorithm requires slightly more iterations compared to the original FIIB algorithm. This phenomenon can be explained by the reduction in computational redundancy in the improved algorithm. The original FIIB algorithm processes the entire spectrum, while the improved FIIB algorithm iterates only in the positive frequency half-band. At low SNR, noise dominates the spectrum, and the reduced redundancy makes the optimization process more sensitive to the variance of the noise. Although the improved FIIB algorithm requires slightly more iterations than the original, the difference in the number of iterations never exceeds two. As the SNR increases (SNR>4dB), the iteration counts of the two algorithms converge, demonstrating that the improved FIIB maintains favorable convergence stability under common SNR conditions.

**Figure 14:**
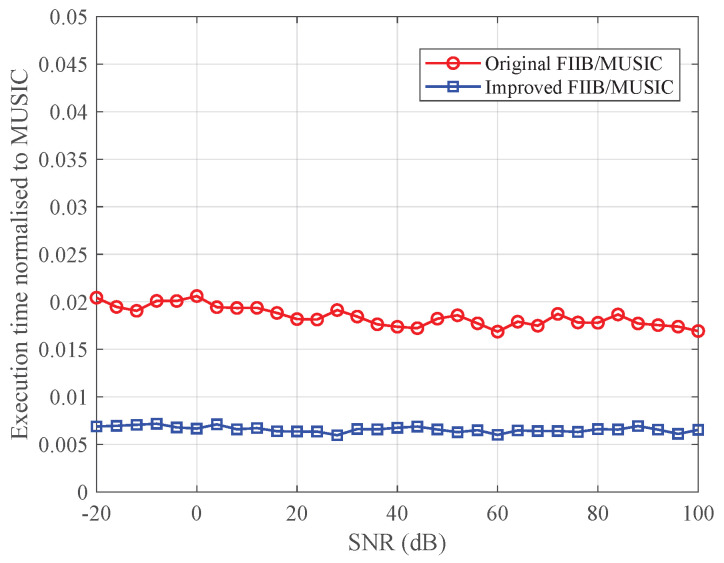
Ratio of execution time of the improved and original FIIB to the MUSIC versus SNR.

To further evaluate computational efficiency, Figure 14 compares the execution time ratios of the improved and original FIIB algorithms relative to the MUSIC algorithm. It is observed that across the entire SNR range, the computation times of both FIIB algorithms are significantly lower than that of the MUSIC algorithm. More importantly, the improved FIIB algorithm further reduces the runtime by approximately 60%. This enhancement is primarily attributed to two key optimizations. First, during the iterative cancellation of the spectral leakage from previously estimated components, the conjugate symmetry of real-valued signals is leveraged to use twice the real part of the positive frequency component’s leakage to equivalently replace the combined leakage from both positive and negative frequency components, thus avoiding redundant calculations. Second, the frequency search is constrained to the positive frequency band, requiring the estimation of only half the number (L/2) of targets. These results consistently demonstrate that the proposed improved FIIB algorithm effectively reduces the computational burden without compromising frequency estimation accuracy or convergence stability, making it more suitable for real-time vital sign monitoring applications.

## 4. Experimental Results and Analysis

### 4.1. Radar and Measurement Metrics

#### 4.1.1. Radar Introduction and Parameter Selection

**Figure 15:**
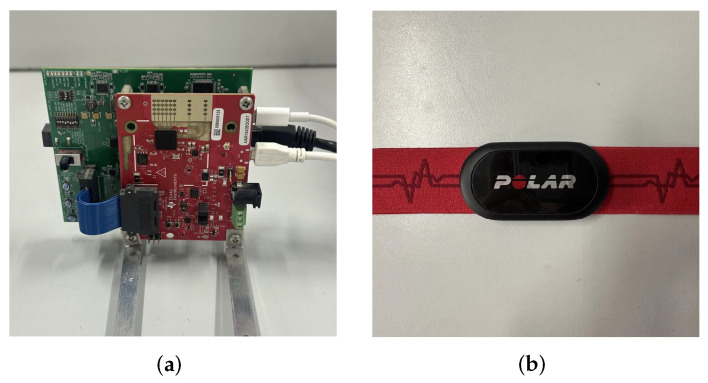
Experimental equipment. (**a**) AWR1642 radar. (**b**) Wearable heart rate sensor Polar H10.

In the experiment, we used the Texas Instruments millimeter-wave FMCW radar (AWR1642, Texas Instruments, Dallas, TX, USA [50]), which is shown in Figure 15a. This study used a 2×4 TDM-MIMO configuration, and the radar’s detailed parameters are listed in Table 1. Each set of experimental data contains 1200 frames, corresponding to 60 s. To ensure the update rate of the HR, the data are processed using overlapped sliding windows of 25.6 s with 24.6 s of overlap, which results in updated estimates every Δt=1s. In addition, the specific parameter settings for the proposed algorithms (including Two-stage accumulation, HDBS, and VLW) are summarized in Table 2. These parameters were optimized based on preliminary experiments to balance performance and computational efficiency.

#### 4.1.2. Experimental Design and Setup

The experiments involved 10 healthy volunteers (Age: 20–30 years; BMI: 18.5–24.0 kg/m^2^; Gender: 5 males and 5 females). The study was conducted in typical laboratory and hall environments with ambient temperatures ranging from 5 °C to 25 °C and a relative humidity of approximately 40–60%. To simulate realistic application scenarios, common sources of electromagnetic interference, such as Wi-Fi routers and computers, were intentionally retained.

The radar was mounted on a desk at a height of 0.8 m, ensuring the detection angle was aligned with the subjects’ chest area. During data acquisition, subjects were instructed to sit naturally and breathe normally without specific pacing constraints. The wearable commercial device Polar H10 (as shown in Figure 15b) was used as the reference for true heart rates. To ensure an unbiased evaluation of the algorithm’s robustness, no datasets were excluded from the analysis.

#### 4.1.3. Scheme Evaluation Metrics

We use the relative error to assess the distance estimation accuracy of the HDBS algorithm, adopt the difference between the estimated DOA and the true DOA to evaluate the angle estimation accuracy of the Root-MUSIC algorithm, and use MAE to evaluate the HR estimation accuracy for different schemes. The expression of relative error δ is formulated as Equation (23):

(23) $$\delta =\frac{\hat{d}-d_{0}}{d_{0}}\times 100$$

where $\hat{d}$ is the measured value, and $d_{0}$ is the true value. In addition to the MAE, the standard deviation (SD) is utilized to evaluate the stability and robustness of the estimation across different experimental scenarios. It is calculated in Equation (24) that

(24) $$e_{\mathrm{SD}}=\sqrt{\frac{1}{N_{r}-1}\sum_{n=1}^{N_{r}}{\left(E_{n}-\bar{E}\right)}^{2}}$$

where $N_{r}$ denotes the total number of independent experiments, $E_{n}$ represents the MAE for the *n*-th dataset, and $\bar{E}$ is the average MAE across all $N_{r}$ datasets.

### 4.2. Single-Target Experiment

**Figure 16:**
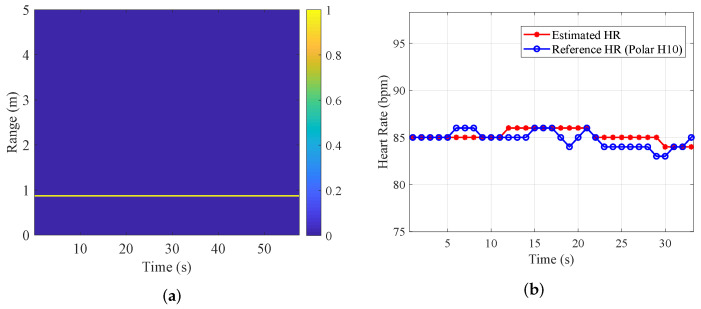
Single-target performance. (**a**) Range-time map. (**b**) Estimated HR compared with true value.

Before evaluating the system’s performance in complex multi-target scenarios, it is essential to establish its baseline performance on a single subject. A specific experiment was conducted with a stationary subject located approximately 0.9 m from the radar. Figure 16 illustrates the processing results, providing a detailed visualization of the target localization and heart rate tracking capabilities. Figure 16a presents the Range-Time Map derived from the HDBS algorithm. A distinct and continuous target trajectory is clearly visible, confirming that the target at 0.9 m is accurately localized. Figure 16b compares the HR estimated by the proposed framework (red curve) against the reference ground truth provided by the Polar H10 sensor (blue curve) over a continuous observation window. The proposed method exhibits excellent tracking capability, with the estimation curve tightly aligning with the reference even during minor HR fluctuations.

To rigorously validate the accuracy, the proposed framework was compared with the conventional single-target HR estimation method (VLW decomposition combined with standard FFT). We conducted ten independent trials to ensure statistical reliability. The traditional FFT-based method yielded an average MAE of 2.42 bpm. In contrast, the proposed VLW-FIIB framework achieved a significantly lower average MAE of 0.95 bpm. Additionally, HDBS achieved an average relative distance error of 1.02%. These results establish a solid baseline for subsequent multi-target separation tasks.

### 4.3. Experiment Involving Two Subjects

#### 4.3.1. Subjects Are at Different Range Bins but at the Same Azimuth Angle

**Figure 17:**
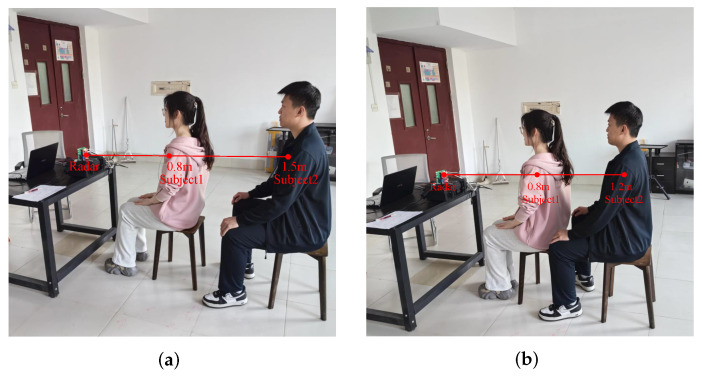
Two experimental scenarios. (**a**) Two subjects were far apart in front and behind. (**b**) Two subjects were close together in front and behind.

In these experiments, two kinds of multi-target occlusion scenarios are constructed to verify the discriminative capability of the proposed scheme. As illustrated in Figure 17, all subjects were positioned along the radar’s normal direction, with subject 2 obscured by subject 1. In Figure 17a, two subjects were positioned 0.8 m and 1.5 m from the radar (0.7 m apart). In Figure 17b, their spacing was reduced to 0.4 m.

Table 3 tabulates the distance, angle, and HR estimation errors across five datasets under two scenarios. Herein, VLW+FIIB constitutes the proposed scheme in this paper. By comparing this scheme with VLW+FFT (where only FIIB is replaced by FFT, with all other preceding operations remaining consistent), the performance improvement by adopting FIIB can be distinctly observed. Table 4, Table 5 and Table 6 follow the same principle and will not be explained separately.

In Table 3, the relative error in distance estimation exhibits a maximum of 2.81%, a minimum of 0%, and an average of 1.72%. In DOA estimation, the angular error ranges from 0.29° to 1.56°, with an average error of 0.77°. These performances also benefit from the accumulation gain achieved, respectively, in distance and angle estimation. These results demonstrate that the proposed scheme effectively discriminates multiple targets even under occlusion conditions. After the VLW algorithm suppresses interference and extracts high-quality heartbeat signals, the average MAEs of the HR estimation by FIIB and FFT are 2.54 bpm and 4.05 bpm, respectively. Remarkably, adopting the FIIB algorithm achieves a performance improvement of 1.51 bpm. This is primarily attributed to FIIB’s capability for high-precision and high-resolution frequency measurement. These results strongly confirm the excellent accuracy and effectiveness of the proposed scheme based on VLW and FIIB in HR estimation. Furthermore, the lower standard deviation of the proposed scheme (0.67 bpm) further demonstrates that, compared to the traditional FFT method (1.00 bpm), the FIIB algorithm exhibits superior robustness and stability across different subjects and heart rate ranges.

#### 4.3.2. Subjects Are at the Same Range Bin but at Different Azimuth Angles

**Figure 18:**
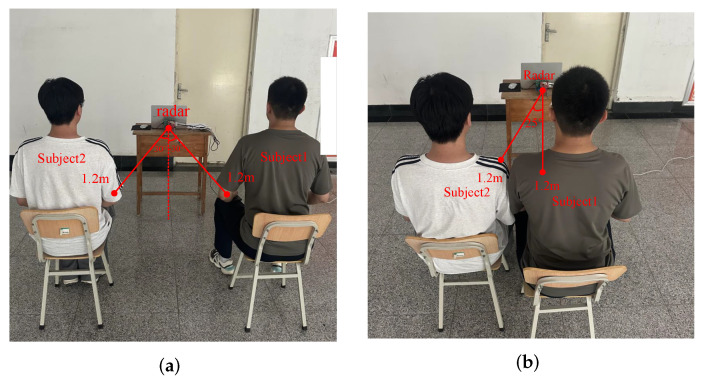
Two experimental scenarios. (**a**) 60° azimuth spacing. (**b**) 25° azimuth spacing.

Figure 18 illustrates the experiment on the proposed scheme’s performance when subjects were at the same distance. Specifically, Figure 18a showed an angular spacing of 60° between two subjects, while Figure 18b showed a spacing of 25°.

Table 4 lists estimation errors of distance, angle, and HR estimation for five datasets in two scenarios. The relative error of distance varies from 0.87% to 1.63%, with an average of 1.30%, while DOA errors range from 0.07° to 1.53°, with an average of 0.69°. These results demonstrate that two subjects can be discriminated in both scenarios, even if they sit closely side by side. In HR estimation, the FIIB algorithm achieves an average MAE of 1.91 bpm, yielding a 1.2 bpm improvement compared to FFT, further demonstrating the proposed scheme’s ability in effectively separating multiple targets and accurately estimating heart rate.

#### 4.3.3. Subjects Are at Different Range Bins and Azimuth Angles

**Figure 19:**
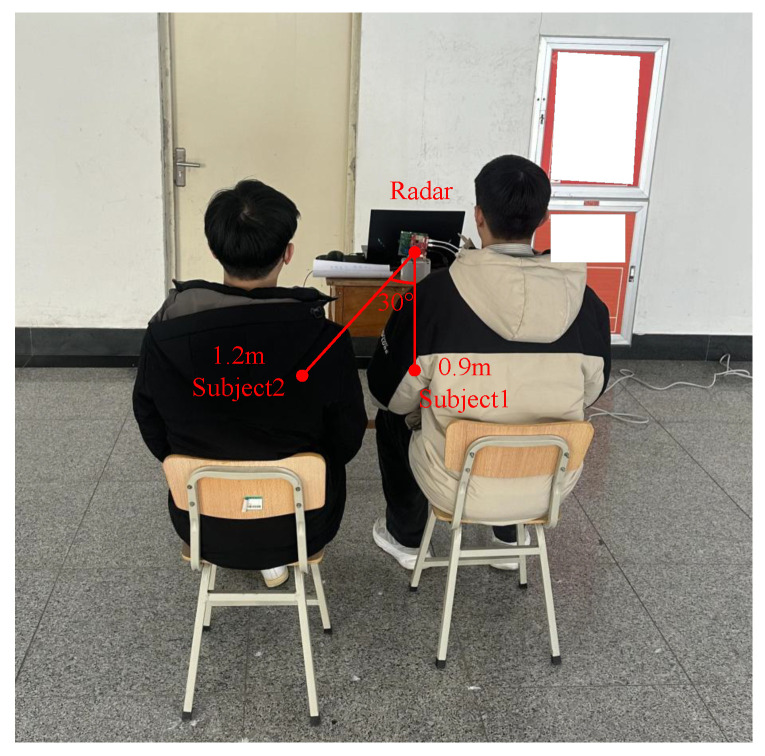
Experimental scenarios with two subjects located at different range bins.

In Figure 19, targets were close without occlusion, so multi-target discrimination can be achieved by using range bin information or applying Root-MUSIC. This experimental design is simpler compared to Section 4.3.1 and Section 4.3.2, so only a brief description of the results is given.

As shown in Table 5, the average relative error of distance is 1.31%, the average error of DOA is 0.54°. The average MAE of the HR estimation using FIIB is 1.02 bpm, and an improvement of 1.17 bpm can be attained compared to the scheme using FFT. It is worth noting that while the FFT method exhibits a slightly lower inter-experiment SD (0.37 bpm), its significantly higher MAE suggests a systematic bias likely caused by the grid-limited frequency resolution. In contrast, the FIIB algorithm provides super-resolution estimation that fluctuates closely around the true value, resulting in a much lower mean error. It is evident that the proposed scheme can also achieve multi-target discrimination and high accuracy of HR estimation in this case.

### 4.4. Experiment Involving Three Subjects

**Figure 20:**
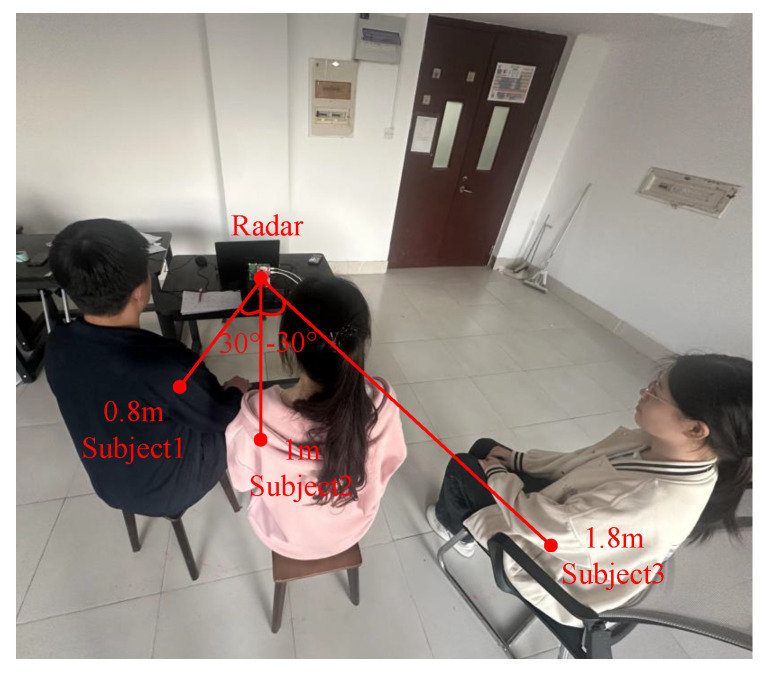
Experimental scenario with three subjects.

A third subject was added to the experiment to emulate the scenario of three people randomly seated in a room. As shown in Figure 20, subjects 1–3 were 0.8 m, 1 m, and 1.8 m from the radar with azimuth angles of 30°, 0°, and −30°, respectively. From Figure 16, subjects 1 and 2 were deployed with smaller spatial separation, and their results more effectively demonstrate the performance of the proposed scheme in the scenario with three subjects. Therefore, the detailed results for subjects 1 and 2 are listed in Table 6, which shows that the average relative error of distance is 1.92%, the average error of DOA is 0.70°. For HR estimation, the FIIB algorithm yields an average MAE of 1.88 bpm, with a 1.28 bpm improvement compared to FFT. Notably, the proposed scheme can effectively discriminate multiple subjects and achieve high accuracy of HR estimation in the scenario with three subjects.

### 4.5. Comprehensive Analysis of Experimental Results

The average relative error of distance in single-target scenarios is 1.02%, while it reaches 1.65% in multi-target scenarios. In comparison to single-target scenarios, the mutual interference between multiple targets increases the relative error of distance.

For HR estimation, the proposed scheme is evaluated under various scenarios. In single-subject, two-subject, and three-subject scenarios with no occlusion between subjects, the average MAEs are 0.95 bpm, 1.46 bpm, and 1.88 bpm, respectively. As the number of targets increases, the mutual interference between subjects becomes more pronounced, causing a decrease in HR estimation performance of the proposed scheme, but the decrease is not significant. When there is occlusion between two subjects, an average MAE increases further to 2.54 bpm, which still represents acceptable performance.

**Figure 21:**
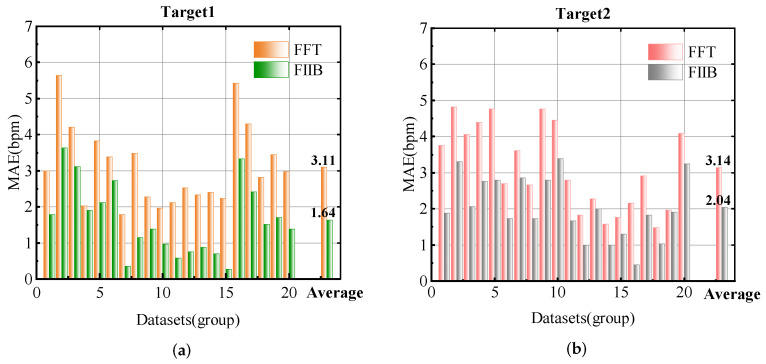
MAE of HR estimation for all multi-target scenarios. (**a**) Target1. (**b**) Target2.

In our proposed scheme, the VLW algorithm effectively suppresses signal interference while extracting high-quality heartbeat signals, and an improvement of 1.7 bpm in MAE can be attained compared with the traditional VMD method, establishing a robust foundation for accurate HR estimation. Figure 21 presents the comparison results of MAE for two-target HR estimation using FIIB and FFT in all multi-target scenarios. The results show that compared with the traditional FFT scheme, the scheme using FIIB improves the average MAE of two targets by 1.29 bpm. Therefore, VLW and FIIB constitute an effective combination in our proposed multi-target HR estimation scheme. This scheme exhibits robust performance in complex environments, achieving an average MAE of 1.84 bpm for targets in all multi-target scenarios.

**Figure 22:**
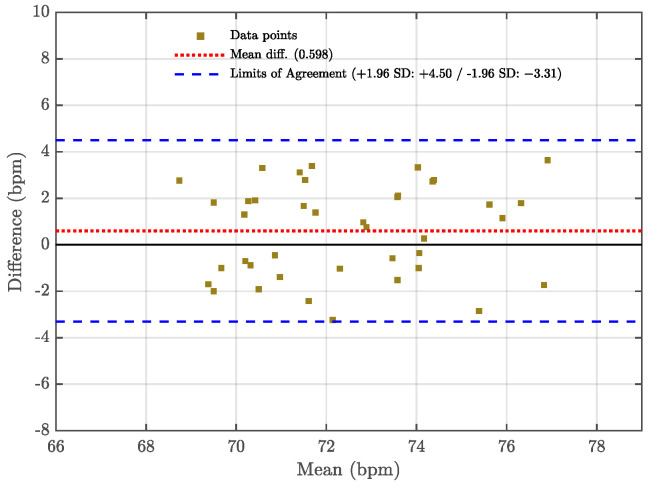
Bland–Altman Experimental analysis of all HR estimation results.

To statistically validate the reliability of this performance improvement, a paired *t*-test was conducted on the 20 datasets across all experimental scenarios. The results confirm that the proposed FIIB algorithm achieves a statistically significant reduction in MAE compared to the FFT method (p<0.001). Furthermore, to assess the clinical agreement between the radar system and the Polar H10 reference device, a Bland–Altman analysis was performed on all 20 datasets, as illustrated in Figure 22. The analysis reveals a mean bias of 0.598 bpm, with 95% limits of agreement (LOA) ranging from −3.31 to 4.50 bpm. The narrow LOA indicates a high level of agreement between the radar measurements and the contact-based sensor, verifying the reliability of the proposed method for vital sign monitoring.

### 4.6. Comparison with Recent Literature

Table 7 presents a comparison of state-of-the-art methods for multi-subject vital sign detection. In Table 7, “Dif. ranges Dif. angles” refers to scenarios in which targets are located at different range bins and azimuth angles.

First, existing studies exhibit limitations in scenario coverage. Specifically, references [11,12] only achieve multi-target localization without further separating targets to estimate HR. References [15,27] solely focus on scenarios with multiple targets at the same distance, lacking the analysis of other complex scenarios. Eder et al. [13] rely on the distance dimension for subject localization, which is not applicable when multiple targets are at the same distance from the radar. References [14,16,20,25] address scenarios with multiple targets in the same range bin, but overlook scenarios involving occlusion between subjects. Reference [26] can deal with slight occlusion cases, but their performance in complex occlusion environments remains unanalyzed.

Second, it is crucial to acknowledge that a direct numerical comparison is limited by differing experimental conditions. For instance, the results in [15] represent classification accuracy rather than continuous tracking error (MAE). More importantly, references [13,14] exhibit lower HR estimation errors, primarily attributed to their experiments being conducted under ideal conditions (SNR=0dB).

In contrast, the results of this work (MAE<2.6bpm) were obtained in six complex physical scenarios, including front-back occlusion and low signal strength. While some baseline methods achieve lower errors in ideal environments, our proposed framework demonstrates superior robustness and applicability in realistic, non-line-of-sight monitoring environments.

### 4.7. Computational Complexity and Real-Time Feasibility

To assess the potential for embedded deployment, we conducted a comprehensive analysis of the computational complexity and resource consumption based on real-world experimental data. As detailed in Section 4.1.1, the system operates using a sliding window mechanism to ensure continuous monitoring. To rigorously evaluate the computational burden, we measured the average execution time per sliding window across the complete 60-s experimental dataset on the evaluation platform using MATLAB (version R2018a). Table 8 summarizes the theoretical algorithmic complexity (Big-O notation) and the measured execution times for the core modules.

In terms of time complexity, the HDBS algorithm exhibits high efficiency, requiring only 0.031 s. Although the theoretical complexity of the clustering phase is $O(N_{p}^{2})$, the optimized implementation ensures rapid execution even when processing the Range-Doppler map. Similarly, the Root-MUSIC algorithm demonstrates exceptional speed, requiring only 0.033 s. This is because it abandons the computationally expensive spectral grid search of the traditional MUSIC algorithm in favor of fast polynomial rooting ($O(M^{3})$), and this operation is performed only on detected targets. Regarding signal processing, the VLW module remains the most computationally intensive part (0.270 s) due to the iterative nature of VMD. In stark contrast, the proposed improved FIIB algorithm (O(NlogN)) requires only 0.009 s, its runtime is dominated primarily by the initial FFT operation.

## 5. Conclusions

This paper presents a novel multi-target heart rate detection framework, specifically designed to overcome the challenges of distinguishing targets in closely spaced and occluded scenarios. Furthermore, the scheme achieves high SNR gains through low-computational-cost accumulation strategies in both distance and angle estimation stages.

Regarding target location, the proposed HDBS algorithm exhibits excellent distance estimation performance with an average relative error of 1.56%, enabling precise target identification without prior knowledge of the subject count. Complementing this in the angular domain, the integration of the Root-MUSIC algorithm effectively overcomes the resolution limitations of conventional FFT-based methods, achieving high-precision target discrimination with an average DOA error of 0.68°.

In terms of signal enhancement, the novel VLW technique effectively extracts high-quality heartbeat components via multi-layer decomposition and interference suppression. Compared to conventional VMD, this module independently reduces the heart rate estimation MAE by 1.7 bpm. Finally, to meet the requirements of high precision and super-resolution, the improved FIIB algorithm achieves an accuracy improvement of 1.29 bpm over traditional FFT, with statistical significance confirmed by a paired *t*-test (p<0.001).

Experimental results confirm that the proposed system maintains an average HR MAE below 2.6 bpm across complex scenarios. However, certain limitations and applicability boundaries remain. First, the experimental conditions of this study are relatively idealized. The current framework assumes that targets are stationary (with steady breathing) and facing the radar directly. Consequently, it fails to adequately address common interference factors in practical scenarios, such as slight body movements and abnormal breathing patterns. Second, the impact of physiological variations (e.g., BMI, clothing thickness) has not been exhaustively analyzed. Third, while effective for up to three targets within a short range (less than two meters), the system’s scalability to larger crowds and extended detection ranges requires further investigation. Finally, in highly cluttered environments, factors such as moving background objects or severe multipath effects could potentially generate ghost targets.

Consequently, future work will focus on enhancing the adaptability of the framework to diverse real-world conditions. Specifically, we will investigate advanced signal processing techniques to mitigate the effects of slight body movements and irregular respiration. Simultaneously, advanced beamforming techniques will be investigated to mitigate multipath and coupling effects. Finally, we aim to optimize the algorithm’s architecture for real-time deployment on low-power edge devices, thereby advancing intelligent monitoring in complex environments. 

## Figures and Tables

**Table 1:** Radar parameters.

Parameter	Value
Initial Frequency	77GHz
Frequency Bandwidth	3.99GHz
Sweep Slope	70MHz
ADC Sampling Time	50μs
Fast-time Sampling Frequency	4MHz
ADC Sampling Points	200
Slow-time Sampling Frequency	20Hz
Frame Period	50ms

**Table 2:** Key parameter settings of the proposed signal processing algorithms.

Methods	Parameter	Symbol	Value
Two-stage Accumulation	Time segment length	*K*	128
	Accumulation factor	*P*	8
HDBS	neighborhood radius (DBSCAN)	eps	6 (range bins)
	Min samples (DBSCAN)	min_samples	5
VLW	Decomposition modes (VMD)	$K_{\mathrm{VMD}}$	6
	Penalty factor (VMD)	α	2000
	Correlation threshold (LMD)	$\rho_{\mathrm{LMD}}$	0.35
FIIB	Iteration counts	*Q*	10

**Table 3:** Estimation error with two subjects at different range bins but at the same azimuth angle.

Angle (deg)	Range (m)	Dataset	Subject	Error Metrics			
				Range Error (%)	DOA Deviation (deg)	HR MAE (bpm)	
				HDBS	Root-MUSIC	VLW+FIIB	VLW+FFT
0	0.8 1.5	1	1	0	0.71	1.79	3.00
			2	2.00	0.95	1.88	3.76
		2	1	2.25	0.86	3.64	5.64
			2	2.81	0.90	3.30	4.82
	0.8 1.2	3	1	1.67	0.28	3.12	4.21
			2	2.22	0.36	2.06	4.06
		4	1	0.71	1.56	1.91	2.03
			2	2.08	1.03	2.76	4.39
		5	1	1.67	0.48	2.12	3.82
			2	1.74	0.57	2.79	4.76
**Average**				**1.72**	**0.77**	**2.54**	**4.05**
**SD**				–	–	**0.67**	**1.00**

**Table 4:** Estimation error with two subjects at the same range bin but at different azimuth angles.

Range (m)	Angle (deg)	Dataset	Subject	Error Metrics			
				Range Error (%)	DOA Deviation (deg)	HR MAE (bpm)	
				HDBS	Root-MUSIC	VLW+FIIB	VLW+FFT
1.2	−30 30	1	1	0.87	0.07	2.73	3.39
			2		0.46	1.73	2.70
		2	1	1.29	0.14	0.36	1.79
			2		0.39	2.85	3.61
	0 25	3	1	1.63	0.51	1.15	3.48
			2		1.75	1.73	2.67
		4	1	1.26	0.74	1.39	2.27
			2		0.48	2.79	4.76
		5	1	1.48	1.53	0.97	1.97
			2		0.86	3.39	4.45
**Average**				**1.30**	**0.69**	**1.91**	**3.11**
**SD**				–	–	**0.98**	**1.01**

**Table 5:** Estimation error with subjects located at different range bins and azimuth angles.

Range (m)	Angle (deg)	Dataset	Subject	Error Metrics			
				Range Error (%)	DOA Deviation (deg)	HR MAE (bpm)	
				HDBS	Root-MUSIC	VLW+FIIB	VLW+FFT
0.9 1.2	0 30	1	1	0	0.73	0.58	2.12
			2	0.93	0.36	1.67	2.79
		2	1	1.40	0.23	0.76	2.52
			2	2.13	0.27	1.00	1.82
		3	1	1.67	1.02	0.88	2.33
			2	2.60	0.16	2.00	2.27
		4	1	0.87	0.31	0.70	2.39
			2	1.08	0.78	1.00	1.58
		5	1	1.08	1.03	0.27	2.24
			2	1.31	0.48	1.30	1.76
**Average**				**1.31**	**0.54**	**1.02**	**2.18**
**SD**				–	–	**0.52**	**0.37**

**Table 6:** Estimation error with three subjects located at different range bins and azimuth angles.

Range (m)	Angle (deg)	Dataset	Subject	Error Metrics			
				Range Error (%)	DOA Deviation (deg)	HR MAE (bpm)	
				HDBS	Root-MUSIC	VLW+FIIB	VLW+FFT
0.8 1 1.8	30 0 −30	1	1	1.60	0.22	3.33	5.42
			2	2.31	1.02	0.45	2.15
		2	1	1.76	0.28	2.42	4.30
			2	1.93	0.58	1.82	2.91
		3	1	2.30	0.82	1.52	2.82
			2	2.65	0.15	1.03	1.48
		4	1	1.42	1.54	1.70	3.45
			2	1.78	1.06	1.91	1.97
		5	1	1.08	1.34	1.39	2.97
			2	2.33	1.03	3.24	4.09
**Average**				**1.92**	**0.70**	**1.88**	**3.16**
**SD**				–	–	**0.91**	**1.19**

**Table 7:** Performance comparison of the proposed work with state-of-the-art multi-subject detection techniques.

Ref.	Radar	TX /RX	Dif. Ranges Dif. Angles	Min. Distance Spacing (m)	Sam. Range Dif. Angles	Min. Azimuth Spacing (deg)	Dif. Ranges Sam. Angles	Min. Azimuth Spacing (deg)
			HR Error		HR Error		HR Error	
[11]	CW	8/8	NA	/	NA	/	NA	/
[12]	CW	2/2	NA	/	NA	/	NA	/
[15]	FMCW	3/4	NA	/	Accuracy > 82.89%	30	NA	/
[27]	FMCW	1/1	NA	/	MAE<2.65 bpm	60	NA	/
[13]	FMCW	1/1	MAE=1.18 bpm (SNR=0dB)	0.6	NA	/	NA	/
[14]	FMCW	1/4	MAE<1 bpm (SNR=0dB)	0.52	MAE<1 bpm (SNR=0dB)	60	NA	/
[16]	FMCW	2/4	Accuracy >96%	0.4	Accuracy >96	80	NA	/
[20]	FMCW	1/1	MAE<3.14 bpm	0.2	MAE<3.14 bpm	>70	NA	/
[25]	IR -UWB	1/1	Error=4.11%	0.8	Error=5.02%	NA	NA	/
[26]	CW	4/4	MAE<3 bpm	0.35	MAE<3 bpm	15	MAE<3 bpm	10
This work	FMCW	2/4	MAE=1.45 bpm	0.2	MAE<1.91 bpm	25	MAE=2.54 bpm	0

Note: All values are directly cited from the respective original literature.

**Table 8:** Computational complexity and execution time analysis of key algorithms.

Algorithms	Time Complexity (Big-O)	Execution Time	Notes
HDBS	$O(N_{p}^{2})$	0.031 s	$N_{p}$: Detected candidate points
Root-MUSIC	$O(M^{3})$	0.033 s	*M*: Array dimension
VLW	$O(K_{\mathrm{VMD}}\;\cdot \;N\log N)$	0.270 s	$K_{\mathrm{VMD}}$: VMD modes *N*: Signal length
Improved FIIB	O(NlogN)	0.009 s	*N*: Signal length Dominated by initial FFT
Total System	–	0.343 s	

## Data Availability

The datasets presented in this article are not readily available because they are part of an ongoing study. Requests to access the datasets should be directed to the corresponding authors.
